# Spatial patterns and determinants of undernutrition among late-adolescent girls in Ethiopia by using Ethiopian demographic and health surveys, 2000, 2005, 2011 and 2016: a spatial and multilevel analysis

**DOI:** 10.1186/s12889-021-11959-3

**Published:** 2021-11-04

**Authors:** Nebiyu Mekonnen Derseh, Kassahun Alemu Gelaye, Atalay Goshu Muluneh

**Affiliations:** 1 Department of Internal Medicine, school of Medicine, University of Gondar comprehensive specialized Hospital, University of Gondar, Gondar, Ethiopia; 2 Department of Epidemiology and Biostatistics, Institute of Public Health, College of Medicine and Health Sciences, and comprehensive specialized Hospital, University of Gondar, Gondar, Ethiopia

**Keywords:** Spatial patterns, Undernutrition, Late-adolescent girls, Ethiopia

## Abstract

**Background:**

Undernutrition among late-adolescent girls (15–19 years) in Ethiopia is the highest among Southern and Eastern African countries. However, the spatial variation of undernutrition as a national context is not well understood in Ethiopia. This study aimed at the spatial patterns and determinants of undernutrition among late-adolescent girls in Ethiopia.

**Methods:**

Secondary data analysis was conducted from women’s data of four consecutive Ethiopian Demographic and Health Surveys (EDHS) from 2000 to 2016. A total of 12,056 late-adolescent girls were included in this study. The global spatial autocorrelation was assessed using the Global Moran’s *I* autocorrelation to evaluate the presence of geographical clustering and variability of undernutrition. SaTScan cluster analysis by using the Bernoulli model to detect most likely SaTScan cluster areas of significant high-rate and low-rate of undernutrition was explored. A Multilevel binary logistic regression model with cluster-level random effects was fitted to determine factors associated with undernutrition among late-adolescent girls in Ethiopia.

**Results:**

Undernutrition was clustered nationally during each survey (Global Moran’s *I* = 0.009–0.045, Z-score = 5.55–27.24, *p*-value < 0.001). In the final model, individual and community level factors accounted for about 31.02% of the regional variations for undernutrition. The odds of undernutrition among 18–19 years of adolescent girls, was 57% (AOR = 0.43; 95% CI: (0.35–0.53) lower than those 15–17 years old. Late-adolescent girls with higher educational status were 4.40 times (AOR = 4.40; 95% CI: (1.64–11.76) more likely to be undernourished than those with no educational status. The odds of undernutrition among late-adolescent girls, with the occupation of sales, was 40% (AOR = 0.60; 95% CI: 0.43–0.84) lower than those with not working adolescents. The odds of undernutrition, among late-adolescent girls, having an unimproved latrine type, was 1.79 times (AOR = 1.79; 95% CI: 1.15–2.79) higher than those participants with improved latrine type. The odds of undernutrition among late-adolescent girls with rural residents was 2.33 times higher (AOR = 2.33; 95% CI: 1.29–4.22) than those with urban residents.

**Conclusion:**

Undernutrition among late-adolescent girls was spatially clustered in Ethiopia. The local significant clusters with high prevalence of undernutrition was observed in Northern and Eastern Ethiopia. Those regions with a high prevalence of undernutrition should design interventions to combat undernutrition.

## Background

Adolescence is the period of transition between childhood and adulthood [1] and late-adolescent girls are defined as those female adolescents aged 15–19 years [2]. Adolescent girls are vulnerable to undernutrition because of the increased demand for growth, and development, including sexual development, maturation, and the onset of menarche [3]. Adolescent females aged 15–19 years are more affected than women aged 20–49 years for undernutrition in developing and middle-income countries [2]. Undernutrition among late-adolescent girls is the major neglected public health problem in developing countries including Ethiopia. The South Asian region was the highest- burden of undernutrition among adolescent girls in the world [2], followed by the East Asian region [2].

Sub-Saharan Africa was also the affected region with the burden of undernutrition among late-adolescent girls [2]. Ethiopia is the second most-populous country in Africa, next to Nigeria with an increasing burden of household food insecurity which was affected by recurrent drought and land degradation, population pressure, instability, and social conflict; that was linked with adolescent undernutrition [4]. Ethiopia was the first among Eastern and Southern African countries for late-adolescent girls’ undernutrition [5]. Undernutrition among adolescent girls in Ethiopia is the highest among the other African countries and it is continued to be the major public health problem despite the government invested a huge amount of budget and gave intention for agriculture and its products [6]. During 2016, the prevalence of undernutrition among late-adolescent girls in Ethiopia was 29%, whereas the proportion of overweight or obese was minimal (3%) [7]. A local study conducted in Northwestern Ethiopia also remarked that nearly 50% of adolescent girls were chronically malnourished [8].

Undernutrition resulted in many public health problems. It could affect educational attainment, future productivity, and an increased likelihood of infections like TB, pneumonia, and even early death [9]. Evidence showed that undernourished adolescent girls are the upshot of stunted young, and preschool children; again they will continue to become also malnourished mothers in the future who in turn give birth to low-birth-weight babies that continued to become inter-generation cycle [10, 11]. One in three (33%) late-adolescent girl gives birth during her adolescence period that is more likely to be stunted compared to babies born to mothers who are older than 19 years of age [9]. The impact of undernutrition also leads to stillbirths, small-for-gestational-age neonates, complicated delivery, and even maternal death among married late-adolescent girls [7].

Undernourished adolescent girls are often highly vulnerable to multiple micronutrient deficiencies such as iron deficiency anemia, iodine deficiency disorder, vitamin-D and vitamin-A deficiency, all of these contribute to the risk of infectious and chronic diseases that lead to DALYs and premature deaths [3, 12–14]. A study done in rural Ethiopia revealed that 27% of undernourished adolescent girls were anemic and late-adolescents were four times higher than early-adolescents for developing anemia [15].

Therefore, the intervention of adolescent girls’ undernutrition contributes not only to improve their quality of lives, but is also a key target for MCH improvement. It reduces under-five children stunting by 40%, maternal anemia by 50%, LBW by 30% globally [9].

Evidence showed that the causes of malnutrition are diverse and complex, but they are categorized into two dimensions: immediate causes like inadequate diet and diseases; underlying causes such as food insecurity, limited knowledge, local taboos, culture, inadequate health care access, and poor hygiene-sanitation practices [10]. Scholars also reported that age groups, being rural residence, having family size ≥5, parents’ educational status, dietary diversity score, absence of latrine in HH, unprotected water source for drinking, and food-insecure households were determinant factors of under-nutrition among late-adolescent girls [6, 8, 16, 17].

In Ethiopia, despite the increased health care coverage, and the government’s good commitment towards agricultural production and productive safety net programs, undernutrition among adolescent girls is the continued major public health problem [5–7]. A spatial study is useful for exploring the high burden of undernutrition in specific geographical areas within a community in order to design community-based interventions in such areas. Therefore, understanding the spatial patterns and determinants of undernutrition among late adolescent girls is important for evidence-based decision making to improve adolescents, women, and also future childhood nutritional status in Ethiopia. However, the spatial distributions and determinants of undernutrition among late-adolescent girls in the national context are not well understood in Ethiopia. Therefore, this study contributed spatial patterns and associated factors of undernutrition among late-adolescent girls in Ethiopia and it is important to design better interventions at community levels in the country.

## Methods

### The study settings

This was a national-level study that represented 11 regions of urban and rural areas of Ethiopia from data collected Ethiopian Demographic, and Health Surveys (EDHS) 2000 to 2016. EDHS is the comprehensive, and nationally representative survey conducted in Ethiopia since 2000 as a part of the worldwide DHS program every 5 years-interval [7]. Ethiopia is located in the horn of Africa and lies between latitudes between 3° and 15° North, and longitudes between 33° and 48° East. It has a total area of 1,100,000 km^2^. There are nine ethnically, and politically autonomous regional states, and two administrative cities (Addis Ababa, and Dire-Dawa) in Ethiopia. The Regions are subdivided into sixty 68 zones, and then further into 817 districts, which are further divided into around 16,253 Kebels which are the lowest locally administrative units [7]. Ethiopia is a country with great geographical diversity; its topographic features range from the highest peak at Ras Dashen, which is 4550 m above sea level, and the lowest down to the Afar Depression 110 m below sea level. The climatic condition of the country varies with the topography, and the temperature is as high as 47 °C in the Afar depression and as low as 10 °C in the highlands. Ethiopia is an agrarian country and agriculture is the backbone of the national economy [18]. Ethiopian population growth is fast (53.5 million in the 1994 census that is increased to be 114,963,588 in 2020 and under 25 years are 60% [19].

### Study design and period

This was a secondary data analysis of EDHS data, 2000 to 2016 in Ethiopia.

### Population and eligibility criteria

The source population for this study was all adolescent girls, aged 15 to 19 years at the time of each survey in Ethiopia, while the study population was all adolescent girls, aged 15 to 19 years in Ethiopia who were in the selected EAs and included in the analysis. Adolescent girls 15–19 years who were pregnant or postpartum in the first week were excluded from the study because of increasing weight gain during pregnancy.

### Data source and sampling techniques of EDHS data

We accessed the datasets using the website www.measuredhs.com after requesting from the DHS program database. A total of 12, 056 late-adolescent girls (3545 in 2000, 1550 in 2005, 3700 in 2011 and 3261 in 2016) were included in this study. Sampling weights were done to make representations in each survey due to the non-proportional allocation of the sample to different regions and their urban and rural areas and the possible differences in response rates [7]. Each interviewed unit (household and individual) represented a certain number of similar units in the target population [20]. In EDHS 2016, each region was stratified into urban and rural areas which were grouped into 21 sampling strata [7]. Two-stage stratified cluster sampling techniques for EDHS data were used and details of the methodology were presented from each EDHS report. In the first stage; a stratified sample of census enumeration areas (EAs) in the urban and rural areas was selected with complete household listing using systematic probability sampling based on the sampling frame with population and household information from 1994 and 2007 PHC. In the second stage: the selection of households was carried out by equal probability systematic sampling in the selected EAs. In each selected household, late-adolescents were interviewed with an individual questionnaire [5, 7, 21, 22].

#### Study variables

**The outcome variable** was late-adolescent girls’ undernutrition. Undernutrition was defined as one category of nutritional status for which Body Mass Index (BMI) is less than 18.5 kg/m^2^ which is either stunting or underweight (thinness) [23]. The outcome variable was measured by BMI. The effect of covariates on the outcome variable(undernutrition) for the i^th^ adolescent in the j^th^ cluster (yij) is dichotomized as follows:

$$ \mathrm{yij}=\left\{\begin{array}{c}1\ \mathrm{if}\ \mathrm{BMI}<18.5\ \mathrm{BMI}\ \mathrm{kg}/{\mathrm{m}}^{2\Big)}\left(\mathrm{Undernutrition}\right)\\ {}0\ \mathrm{if}\ \mathrm{BMI}\ge 18.5\ \mathrm{BMI}\ \mathrm{kg}/{\mathrm{m}}^{2\Big)}\left(\mathrm{not}\ \mathrm{undernutrition}\right)\end{array}\right. $$

##### Independent variables

Determinant factors of undernutrition were extracted based on the literature review. Individual-level (level-one) factors like socio-demographic, and socio-economic characteristics, and community-level factors (level-two), are considered to be determinants of undernutrition.

#### Individual level factors (level-one)

##### Socio-demographic factors

Age of adolescent girls, educational status of adolescents, literacy, marital status of adolescents, occupation of adolescents, media exposure, religion, age of HH, number of HH members, number of < 5 children.

##### Socio-economic characteristics

Wealth index, source of drinking water, time to get water, type of latrine, toilet facilities shared with other HH, anemia status, type of cooking fuel, khat chewing, alcohol drinking, covered by health insurance.

Wealth index**:** A composite measure of a household’s cumulative living standard that was divided into 5 quantiles which was derived by using principal component analysis [7].

##### Community level factors (level-two)

Region and residence.

#### Data collection and tools

EDHS data were collected through face-to-face interviews by using questionnaires at the individual and household levels. During each EDHS data collection period, adolescent girls aged 15 to 19 years were asked to give important socio-demographic and socio-economic status and maternal characteristics related to adolescent undernutrition in chronological order [5, 7, 21, 22].

#### Data management and analysis

The data extraction, cleaning, recoding, and labeling for further analysis were done using STATA-14 and Microsoft excel. Sampling weights of each variable were done before conducting analysis to restore for the unequal probability of selection between the strata.

#### Spatial analysis

The global spatial autocorrelation was assessed using the Global Moran’s- *I* (Moran’s- *I*) to evaluate the presence of geographical clustering and variability using ArcGIS Version.10.6. A positive value, statistically significant Moran’s Index indicated a geographical clustering for undernutrition, while statistically significant a negative value Moran’s Index showed dispersion, and if it was zero value, it distributed randomly.

The local Getis-Ord spatial statistical tool was used to identify the statically significant hot spot and cold spot areas. Hot spot refers to the occurrence of the high prevalence of undernutrition that clustered together on the map, whereas cold spot refers to the occurrence of the low prevalence of undernutrition that clustered together on the map.

Kriging interpolation method was used to predict a high prevalence of undernutrition from unobserved enumeration areas in Ethiopia.

We explored spatial scan statistics using the Bernoulli probability model to detect local clusters of significant- high rates and low rates of undernutrition using SaTScan 9.6 software. A cluster is reported to be statistically significant when its log-likelihood ratio (LLR) is greater than the Standard Monte Carlo critical value (C.V) in *p*-value < 0.05. The maximum likelihood ratio test statistic showed the most primary cluster compared with the overall distribution of maximum values. The primary and secondary clusters were identified; LLR was assigned, and the p-value was obtained through the Monte Carlo hypothesis testing with 999 Monte Carlo replicates.

#### Multilevel analysis

A Multilevel binary logistic regression model was fitted to identify the possible factors associated with undernutrition among late-adolescent girls in Ethiopia by using STATA-14. We considered using a multilevel model because each interviewed unit (household and individual) is hierarchical and nested to EAs [7]. Therefore, a two-level model was adopted by taking secondary sampling units (individuals and households) as level-one units, and primary sampling units (EAs) as level-two units. The multilevel binary logistic regression model incorporates fixed effects and cluster-specific random effects to account for the within-cluster correlation of clustered data. Therefore, the two-level fixed and random effects logistic regression model was presented as follows [24]:

$$ {\displaystyle \begin{array}{c} Logit\ (Yij)={\beta}_0j+\sum \beta Xi+\Upsilon Zj+\varepsilon j\\ {}{\beta}_0j={\beta}_{0+}\mu j,\mu j\sim N\ \left(0,{\sigma^2}_u\right)\\ {}\varepsilon j={\varepsilon}_0+\varepsilon j,\varepsilon j\sim N\ \left(0,{\sigma}^2\varepsilon \right)\end{array}} $$

In this model, logit (Yij) = ln (Yij / (1- Yij)) is log-odds for undernutrition called ‘the logit link’. The symbol, ‘Yij’ is a probability of undernutrition for an adolescent girl i in any EA, rural/urban region, ‘j’.’ β_0_j’ is the cluster random intercept. ‘εj’ is the residual for each cluster ‘j. _‘_β’ is fixed effect regression coefficients and ‘Xi’s are level-1 predictors and ϒZj are level-2 factors (community-level) in cluster j.

We considered four models to be fitted for multilevel analysis:

The model I: Empty model which has no individual or community level variables; model II**:** adjusted for individual-level variables; model III**:** adjusted for the community level variables, and model IV: adjusted for both the individual and community level variables. Model comparison was done using Akaike’s Information Criterion (AIC) and the model with the smallest value of AIC was selected as the final best-fitted model. Adjusted Odds Ratios (AOR) with their corresponding 95% CI was calculated to identify the determinants of undernutrition with a *P*-value of < 0.05.

In the random-effects model, we computed Intra-class Correlation Coefficient (ICC), Median Odds Ratio (MOR), and Proportional Change in Variance (PCV) statistics for measures of variation between clusters.

The fixed effects model has the only one source of variability (εj, with its variance σ^2^μ), while the random effects model has two components of variabilities, (εj and ε_0_ with variances σ^2^μ and σ^2^ε respectively). These two sources of variability showed the variability between predictors that are in the same group, measured by the within-group variance σ^2^μ, and the variability between observations that are in different groups, measured by the between-group variance σ^2^ε. The proportion of between-group variance (σ^2^ε) to the total variance (σ^2^μ + σ^2^ε) is called intra-class correlation (ICC) [25]. It is calculated using the formula [26]:

$$ {\displaystyle \begin{array}{c} ICC\left(\rho \right)=\frac{\sigma 2\varepsilon }{\sigma 2\varepsilon +\sigma 2\mu}\\ {}{\sigma}^2\mu =\frac{\pi 2}{3}=3.29\kern1.5em \mathrm{within}-\kern0.5em \mathrm{group}\kern0.17em \mathrm{variance}\left({\sigma}^2\mu \right)\end{array}} $$

The ICC quantifies the variation of undernutrition within clusters. The ICC may range from 0 to 1. ICC = 0 showed perfect independence of residuals and the observations do not depend on clusters. However, ICC = 1 or less than one indicates interdependence of residuals i.e., the variation of observations between clusters [26]. The MOR (Median Odds Ratio) is defined as the median value of the odds ratio between the area at highest risk and the area at lowest risk when comparing two individuals from two different randomly selected clusters and it measures the unexplained cluster heterogeneity. The variation is between clusters by comparing two persons from two randomly chosen different clusters. It is calculated as the following formula [26]:

$$ MOR=\mathit{\exp}\sqrt{2}\times VA2\times 0.6745=\exp \left(0.95\times VA\right) $$

VA is the estimated variance of clusters. The MOR is always greater than or equal to 1. If the MOR is 1, there is no variation between clusters. The total variation attributed to individual and cluster level factors at each model was measured by the proportional change in variance (PCV), which is computed as [26]:

$$ PCV=\frac{VA- VB}{VA}\times 100 $$

The VA is the variance of the initial model and the VB is the variance of the model with more terms.

## Results

### Socio-demographic and economic characteristics of participants

During EDHS 2016, more than half 1949 (60%) of late-adolescent girls were from the age group of 15–17 years, and their median age and IQR were 17 and 2.00, respectively. The majority of the study participants, 2513 (78%) were never married. The majority 2569 (80%) of them were from households by unimproved latrine type. Nearly 1022 (32%) of households had unprotected drinking water sources. Among the participants, 455 (14.12%) and 536 (16.63%) were the poorest and the poorer respectively (Table 1).

**Table 1:** Socio-demographic and economic characteristics of respondents included in the EDHS 2016 analysis

Socio-Demographic and Economic characteristics	Weighted frequency	Percent
Age groups		
15–17	1949	60.5
18–19	1273	39.5
Religion		
Orthodox	1374	42.65
Catholic	20	0.63
Protestant	793	24.61
Muslim	1013	31.44
Others	22	0.67
Current marital status		
never married	2513	77.99
Married	546	16.93
living with partner	19	0.59
Widowed	1	0.03
Divorced	96	2.99
Separated	20	0.61
source of drinking water		
protected	2085	64.70
un-protected	1022	31.71
Others	116	3.59
Time to get drinking water		
Less than 30 min	988	30.66
30 min or longer	1367	|42.42
Water on premises	743	23.05
Others	125	3.87
Type of toilet facility		
Improved	534	16.56
Not improved	2569	79.71
Others	120	3.73
Wealth index		
poorest	455	14.12
Poorer	536	16.63
Middle	609	18.90
Richer	687	21.33
Richest	935	29.02

### Community level characteristics of late-adolescent girls’ undernutrition in Ethiopia

Among undernourished late-adolescent girls, the majority (83.89%) of them were living in rural areas of Ethiopia (Table 2).

**Table 2:** Community level characteristics of respondents included in the EDHS 2016

Community variables	not under-nourished Weighted Freq. (%)	under-nourished Weighted Freq. (%)	Total (%)
Residence			
Urban	604 (80.49)	146 (19.51)	750.50 (100)
Rural	1709 (69.14)	763 (30.86)	2471.86 (100)

This study showed that the prevalence of undernutrition among late-adolescent girls had decreased over time in the past 15 years. It had decreased from 36.79% (95% CI: 34.11–39.54) in EDHS 2000 to 28.22, 95% (CI: 26.02–30.52%) in EDHS 2016, but it was increased from 30.86 (95% CI: 27.84–34.04) in 2005 to 34.82% (95% CI: 32.27–37.47) in 2011 (Table 3).

**Table 3:** The prevalence of undernutrition among late-adolescent girls in Ethiopia, EDHS 2000, 2005, 2011 and 2016

Sr.No.	EDHS	Weighted Prevalence of undernutrition%	95% CI
1	2000	36.79	34.11–39. 54
2	2005	30.86	27.84–34.04
3	2011	34.82	32.27–37.47
4	2016	28.22	26.02–30.52

There was a regional variation of undernutrition among late-adolescent girls in Ethiopia over time. In EDHS 2000 the prevalence of undernutrition was higher (greater than the national average) in regions of Afar (38.93%), SNNPR (40.94%), Tigray (42.55%), Amhara (42.72%), Ben-Gumuz (48.16%), and Somali region (54.64%), whereas in regions of Addis Ababa (23.28%), Oromia (31.93%), Harari (33.43%) and Dre-Dawa (34.95%) were lower. Similarly, in EDHS 2005 the prevalence of undernutrition was higher in Somali (57.39%), Tigray (46.43%), Afar (46.34%), Amhara (37.87%), and Dre-Dawa (37.50%) regions, while Addis Ababa (18.15%), Harari (21.95%), Oromia (25.43%) and SNNPR (27.13%) were observed as lower. Likewise, the undernutrition was reported as higher among late-adolescent girls in regions of Tigray (52.31%), Afar (46.74%), Amhara (44.86%), and Somali (42.48%) in EDHS 2011, while Addis Ababa (21.25%), SNNPR (24.33%), Gambela (26.08) and Dire-Dawa (29.22%) were reported as lower. During EDHS 2016, the higher prevalence of undernutrition was observed in the regions of Somali (45.14%), Tigray (43.31%) and Afar (42.99%), Gambela (38.29%) Amhara (34.32%), regions, whereas, SNNPR (17.37%), Addis Ababa (20.35% and Benishangul Gumuz (20.78%) were reported as lower prevalence. Four regions (Tigray, Afar, Amhara, and Somali) had a higher prevalence in the 2000 to 2016 EDHS period, while, Addis Ababa and Oromia were in lower prevalence (Table 4).

**Table 4:** The regional variation of under-nutrition among late adolescent girls over time in Ethiopia (EDHS 2000–2016)

Sr. No	Region	EDHS 2000		EDHS 2005		EDHS 2011		EDHS 2016	
		Under-nutrition%	Not under-nutrition%	Under-nutrition%	Not under-nutrition%	under-nutrition%	Not under-nutrition%	under-nutrition%	Not under-nutrition%
1.	Tigray	42.55	57.45	46.43	53.57	52.31	47.69	43.31	56.01
2.	Afar	38.93	61.07	46.34	53.66	46.74	53.26	42.99	57.01
3.	Amhara	42.72	57.28	37.87	62.13	44.86	55.14	34.32	65.68
4.	Oromia	31.93	68.07	25.43	74.57	30.11	69.89	26.58	73.42
5.	Somali	54.64	45.36	57.39	42.61	42.48	57.52	45.14	54.86
6.	Ben. Gumuz	48.16	51.84	34.18	65.82	30.27	6973	20.78	79.22
7.	SNNPR	40.94	59.06	27.13	72.87	24.33	75.67	17.37	82.63
8.	Gambela	35.84	64.16	31.36	68.64	26.08	73.92	38.29	61.71
9.	Harari	33.47	66.33	21.95	78.05	31.12	68.88	29.35	79.65
10.	Addis Ababa	23.28	76.72	18.15	81.85	21.25	78.75	20.35	79.54
11.	Dire Dawa	34.95	65.05	37.50	62.50	29.22	70.78	28.46	71.54
12.	Total	36.79	63.21	30.86	69.14	34.82	65.18	28.22	71.78

### Spatial distributions of undernutrition among late-adolescent girls in Ethiopia, EDHS 2000, 2005, 2011 and 2016

**Fig. 1:**
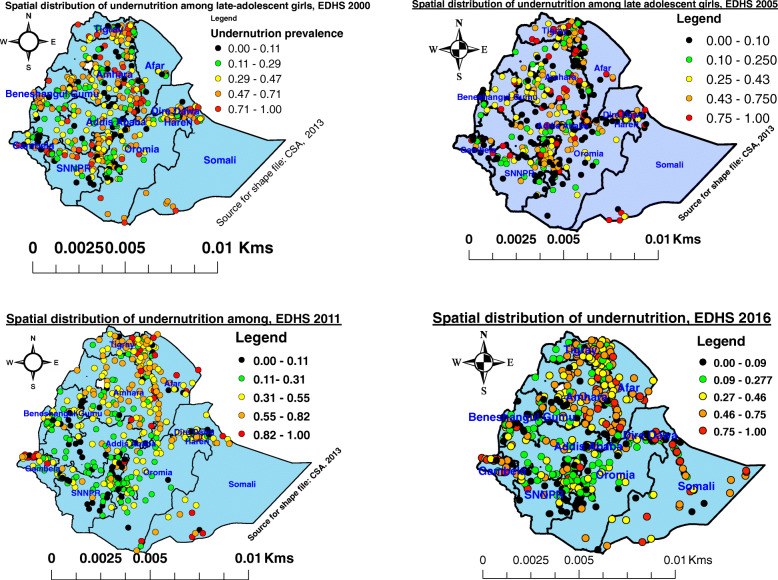
Spatial distribution of late-adolescent girls’ undernutrition in Ethiopia, EDHS 2000 to 2016

During EDHS 2000, the prevalence of undernutrition among late-adolescent girls was spatially distributed with its regional variations. In the figure below (on the left upper side), the red color showed the highest prevalence of undernutrition that was observed in Tigray, Northern and Eastern Amhara, Harari region, Southern Oromia region, SNNPR, Somali region, Gambela, and Benishangul Gumuz regions, while the black color was with the lowest prevalence that covered Central and Southern Tigray, Amhara, Afar, Oromia, SNNPR, Gambela and Benishangul Gumuz, Harari, and Somali regions (Fig. 1 A);

Similarly, in the EDHS 2005, the highest prevalence of undernutrition was observed in the Tigray, Eastern and Southern Afar, Eastern Amhara region, Oromia, Addis Ababa, SNNPR, Harari, and Benishangul Gumuz, Gambela, and Somali region, while the lowest prevalence was distributed all of the regions in the country. The black color indicated the lowest, whereas the red color showed the highest (Fig. 1 B); In EDHS 2011, the highest prevalence (red color) was determined in the regions of Eastern and Southern Tigray, Afar, Eastern Amhara, Northern SNNPR, Harari, Gambela, and Somali region, whereas the low prevalence (black color) was distributed throughout all of the regions, (Fig. 1 C);

Likewise, EDHS 2016 showed that the highest prevalence (the red color) of undernutrition was observed in Southern Tigray, Afar, Eastern Amhara, Gambela, Somali region, and Harari region, while the Southern Amhara region, Oromia, Benishangul Gumuz, SNNPR, and, Addis Ababa were in the lowest prevalence of undernutrition among late-adolescent girls (Fig. 1, D).

Each point data on the map below represents one cluster that showed a prevalence of undernutrition. The prevalence of undernutrition was persistent over 15 years in regions Tigray, Afar, in the Northern and Eastern Amhara, Somali region, and Gambela regions.

### Global spatial autocorrelation analysis (Moran’s *I*) of undernutrition

**Fig. 2:**
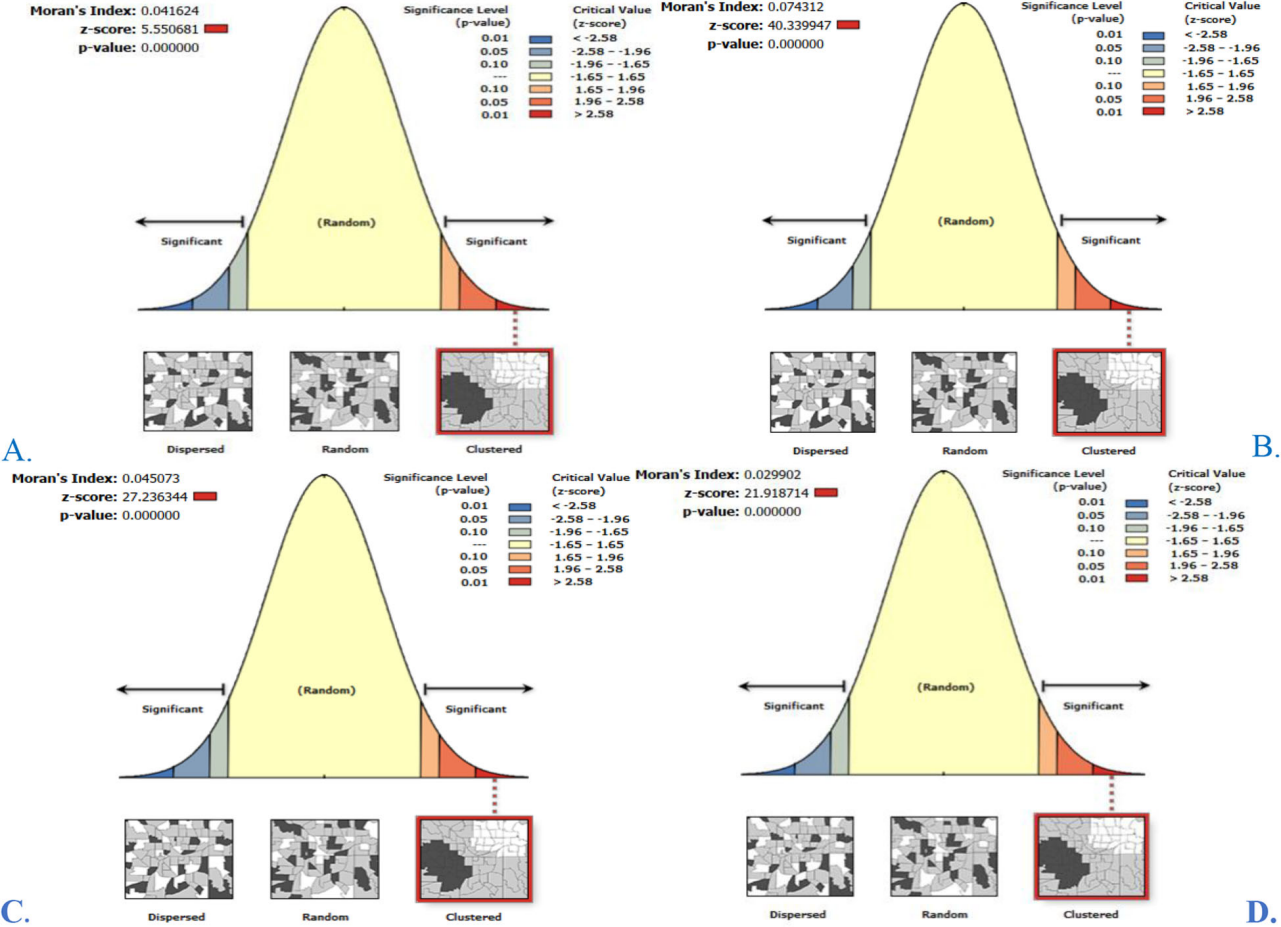
Spatial patterns of undernutrition among late-adolescent girls in Ethiopia (2000, 2005, 2011 and 2016). The clustered patterns showed high rates of undernutrition over the study area and the likelihood of occurrence by random chance are less than 1%

The spatial patterns of undernutrition among late-adolescent girls in Ethiopia were not random in each EDHS period. The global spatial autocorrelation analysis of each survey showed that there were significant clustered patterns of undernutrition across the country (Global Moran’s *I* = 0.042, Z-score = 5.55, *p*-value < 0.001 in EDHS 2000; Global Moran’s *I* = 0.009, Z-score = 5.94, *p*-value < 0.001 in EDHS 2005; Global Moran’s *I =* 0.045, Z-score = 27.24, *p*-value < 0.001 in EDHS 2011 and Global Moran’s *I* = 0.030, Z-score = 21.92, *p*-value < 0.001 in EDHS 2016). This means that undernourished late-adolescent girls with similar patterns depended on one another. Generally, in each output, the Z-score is high and positive with a highly significant *p*-value which showed 99% confidence for clustering of undernutrition across regions in Ethiopia (Fig. 2 A-D). The figures below show that the clustered patterns (on the right side) of high rates of undernutrition across regions in Ethiopia. The bright red and blue colors (to the right and left side) indicated an increased significance level for which the likelihood of clustered patterns occurred by random chance was less than 1% (Fig. 2 A-D).

### Hot spot analysis (Getis-Ord Gi*) of undernutrition among late-adolescent girls in Ethiopia, EDHS 2000 to 2016

**Fig. 3:**
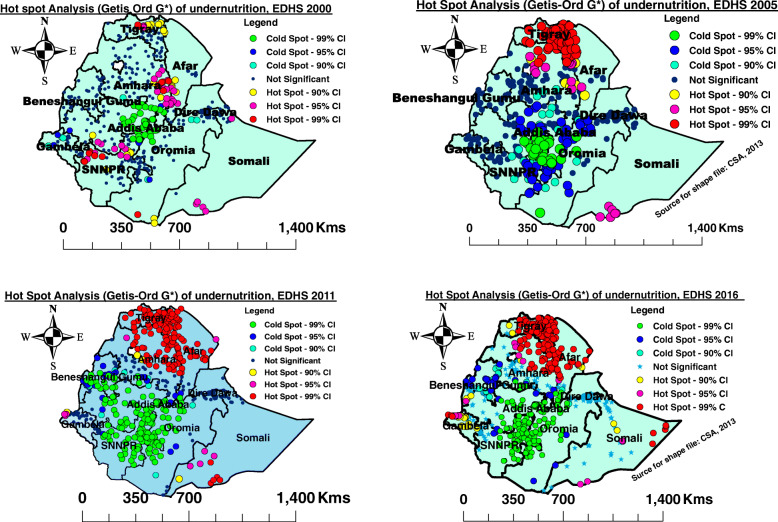
Hot spot analysis of undernutrition among late-adolescent girls in Ethiopia, 2000, 2005, 2011 and 2016

The hot spot areas of undernutrition among late-adolescent girls were observed in the regions of central and eastern Tigray, Southern Afar, Eastern Amhara, Southwest, and Southern Oromia, Southern and Northern Somali region, and SNNPR, whereas Addis Ababa, central Oromia, Southern Amhara region, Northern SNNPR, Harari, and Gambela region were reported as clod spots during EDHS 2000 (Fig. 3 A). Similarly, in 2005, the hot spot areas of undernutrition were observed in regions of Tigray, Western borders of Afar, Northern, and Eastern Amhara, and Southern Somali region, while the cold spot areas were seen in the Southern parts of Amhara region, Oromia region, SNNPR, Benishangul Gumuz, and Addis Ababa (Fig. 3 B).

Likewise, in 2011, the hot spot areas of undernutrition among late-adolescent girls were identified in Tigray, Western and Eastern Afar, Northern, and Eastern Amhara, Southern Somali region, and Gambela regions, while the cold spot areas were seen in Southern Amhara, Southern Afar, Oromia, Addis Ababa, Benishangul Gumuz and SNNPR region (Fig. 3 C). During EDHS 2016, statistically significant hot spot areas were observed in regions of Tigray, Northern and Eastern Amhara region, Afar, Eastern Somali region, and Gambela. The statistically significant cold spot areas were observed in regions of Southern Amhara, Oromia, SNNR, Benishangul Gumuz, Southern Afar, and Addis Ababa (Fig. 3 D). From the figure below, red, pink, and yellow colors showed significant clusters of high risk (hot spot) areas of undernutrition, while green and blue colors showed significant clusters of low risk (cold spot areas).

### Cluster and outlier analysis (Anselin local Moran’s I) of undernutrition among late-adolescent girls in Ethiopia, 2000, 2005, 2011 and 2016

**Fig. 4:**
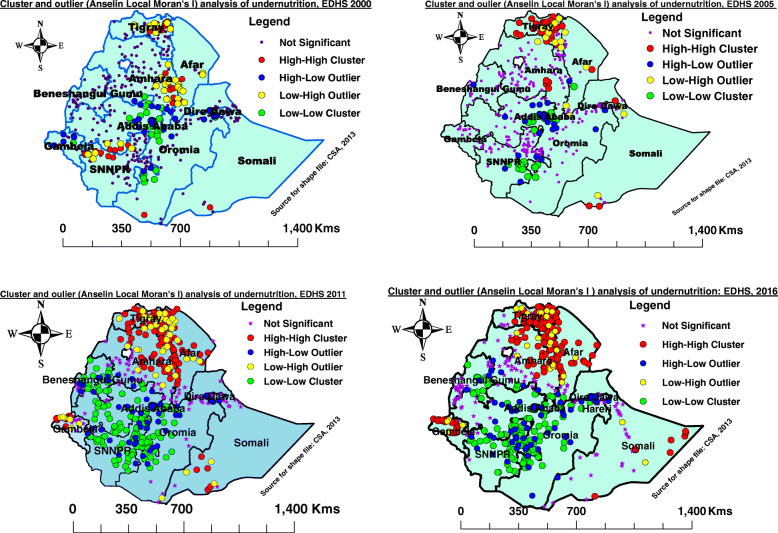
Cluster and outlier analysis of undernutrition among late-adolescent girls in Ethiopia, EDHS 2000 to 2016

During EDHS 2000, statistically significant high-high local clusters (high rates of undernutrition) were observed in Tigray, Southern Afar region, Eastern Amhara, Southwest, and Southern Oromia, Somali region, and Northern SNNPR region, whereas the local low-low clusters were observed in Southern Amhara, Addis Ababa, Northern and Southern Oromia, Northern SNNPR, Gambela, and Harari region. The significant outliers such as low-high happened in central and Eastern Tigray, Afar, Eastern Amhara, Southwest Oromia, and SNNPR, while high-low outliers were observed in Southern Amhara, Northern and Eastern Oromia, Addis Ababa, Dire-Dawa, Harari, and Gambela regions (Fig. 4 A).

Likewise, EDHS 2005 showed that the significant high-high clusters were observed in Tigray, Eastern Amhara region, Eastern Afar, Dire-Dawa, and Northern and Southern Somali region, while the low-low clusters occurred in Oromia, Addis Ababa, SNNPR, and Benishangul Gumuz regions. The low-high outlier was seen in Eastern and Southern Tigray, Northern and Eastern Amhara, Eastern Afar, and Somali region, whereas high-low clusters were seen in Addis Ababa, Northern and Eastern SNNPR, Harari, Benishangul Gumuz and Gambela (Fig. 4 B).

The local cluster and outlier analysis of EDHS 2011 revealed that the significant high-high significant clusters were identified in Tigray, Amhara, Afar, Gambela, and Somali region, whereas the low-low clusters occurred in Addis Ababa, Oromia, SNNPR, Harari, and Benishangul Gumuz regions. The low-high outliers were observed in Tigray, Afar, Eastern Amhara, Gambela, and in the Southwestern Somali region, while Addis Ababa, Oromia, SNNPR, Harari, Dire-Dawa, and Benishangul Gumuz were reported as high-low outliers. (Fig. 4 C). Similarly, EDHS 2016 showed that the high-high significant cluster areas were observed in regions of Tigray, Northern and Eastern Amhara, Afar, Eastern Somali, and Gambela regions, whereas the low-low significant clusters were observed in Southern Amhara, Southern Afar, Addis Ababa, Harari, Oromia, SNNPR and Benishangul Gumuz regions. The low-high outliers were observed in the Tigray region, Afar, Northern and Eastern Amhara, Somali region, and Gambela regions, while Addis Ababa, Dire-Dawa, Harari, Southern Amhara, Oromia region, SNNPR, and Benishangul Gumuz regions were reported as high-low outliers (Fig. 4D). From the figure below, High-High means the high prevalence of undernutrition surrounded by high rates; High-Low means the high prevalence of undernutrition surrounded by low rates of undernutrition; Low-High means the low prevalence of undernutrition surrounded by high rates. Low-Low means low rates of undernutrition surrounded by low rates.

### Spatial interpolation of undernutrition among late-adolescent girls in Ethiopia

**Fig. 5:**
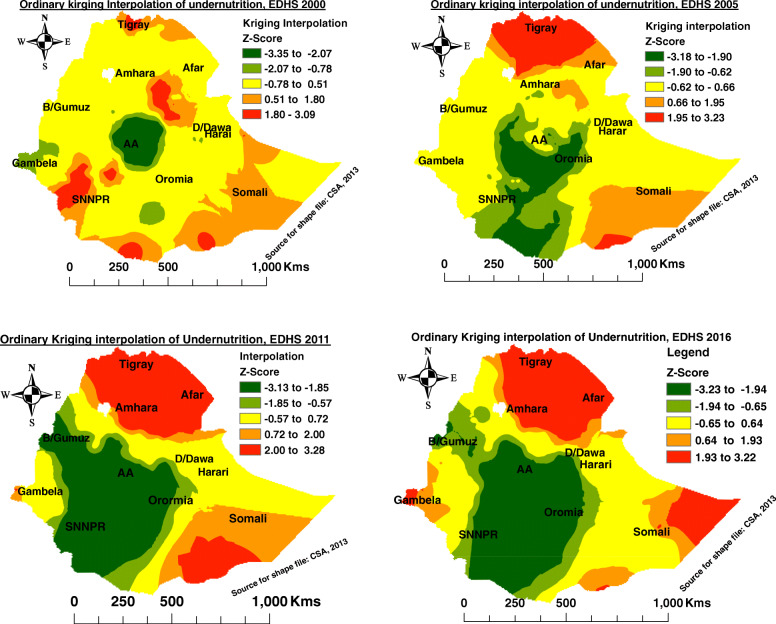
Ordinary kriging interpolation of undernutrition among late-adolescent girls in Ethiopia, EDHS 2000–2016

Ordinary Kriging interpolation in EDHS 2000 showed that there was the highest prediction of undernutrition from un-sampled areas among late-adolescent girls in the regions of Tigray, Afar, Eastern Amhara, Southwestern, and Southeastern Oromia, Southwestern Somali, and NNPR, while the lowest rate of undernutrition was predicted in Addis Ababa and its borders of Oromia and some parts of the Gambela region. From the figure below the red and orange color represented the highest predicted risk while the green color indicated the lowest rates of prediction (Fig. 5 A).

However, EDHS 2005 revealed that the highest prediction of undernutrition among late-adolescent girls from un-sampled enumeration areas were observed in the regions of Tigray, Northern Afar, Northern & Eastern Amhara region, and Southern Somali region. The lowest rates of undernutrition during EDHS 2005 were observed in Addis Ababa, Oromia region and SNNPR (Fig. 5 B).

Similarly, during EDHS 2011, the highest risk of undernutrition was predicted in the un-sampled EAs of Tigray, Afar, Amhara region, Southern Somali and parts of the Gambela regions, while, the lowest rates of undernutrition occurred in Addis Ababa, Oromia, SNNPR, and Benishangul Gumuz regions among late-adolescent girls (Fig. 5 C).

Likewise, during EDHS 2016, the highest prediction of undernutrition risk in the un-sampled enumeration areas was identified in Tigray, Afar, Amhara, Gambela, and Somali region, while the lowest risk of undernutrition was noticed in Addis Ababa, Southern and Southwestern Amhara, most parts of the Oromia region, SNNPR, and Benishangul Gumuz regions among late-adolescent girls (Fig. 5 D).

### Spatial scan statistical analysis of undernutrition among late-adolescent girls in Ethiopia, EDHS 2000 to 2016 using SaTScan software 9.6

**Fig. 6:**
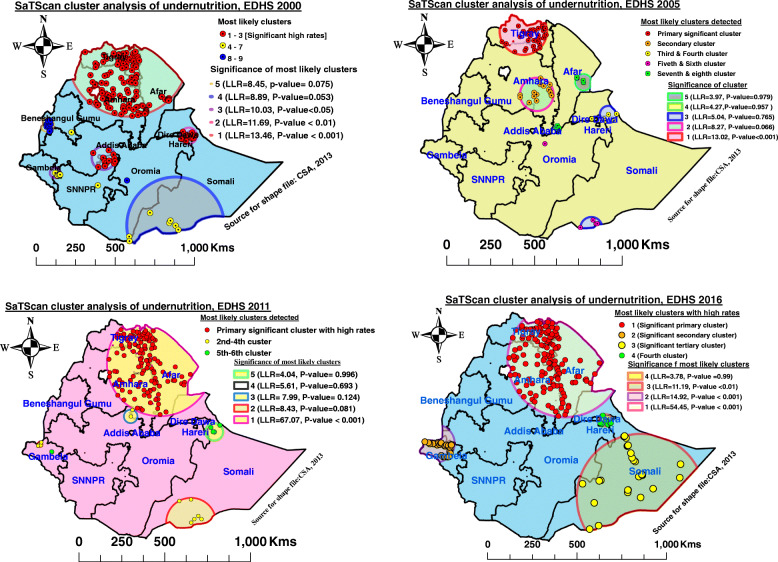
SaTScan cluster analysis of undernutrition among late-adolescent girls in Ethiopia, 2000, 2005, 2011 and 2016

A total of 153 significant clusters, were identified in three most likely clusters during EDHS 2000. The primary significant big cluster of spatial windows encompassed Tigray, most parts of Afar, and Northern and Eastern Amhara regions. It was located at 13.883741 N, 39.162985 E, and 364.77 km radius. Clusters in the primary windows were 1.31 times higher risk of undernutrition than those outside the window (RR = 1.31, LLR = 13.46, *P*-value < 0.001). The primary small significant window included Harari and Northern Somali region, which was centered at 9.506756 N, 42.621090 E and 51.27 km radius. It was 1.73 times higher risk of undernutrition than outside the windows (RR = 1.73, LLR = 11.69 *P*-value < 0.01. The second spatial window covered Southwest Oromia, and Northern borders of SNNPR that was located at 8.245203 N, 37.785581 E, and 74.06 Km radius. Clusters in the second widow was 1.59 times higher risk than outside this window RR = 1.59, LLR = 10.03, *P*-Value < 0.05), respectively (Table 5 and Fig. 6 A).

**Table 5:** Significant clusters of SaTScan analysis for under-nutrition among late adolescent girls in Ethiopia, EDHS 2000 to 2016

Year	Cluster	Significant EAs (clusters) detected	Coordinates /Radius	Population	Cases	RR	LLR	***P***-value
**2000**	Primary	11, 29, 12, 14, 22, 28, 23, 13, 30, 21, 45, 16, 25, 27, 19, 49, 33, 46, 50, 18, 20, 31, 15, 47, 17, 26, 34, 24, 32, 35, 6, 37, 2, 1, 44, 36, 38, 145, 48, 4, 40, 84, 39, 42, 5, 43, 7, 146, 41, 85, 87, 10, 102, 8, 9, 92, 93, 103, 104, 88, 105, 108, 97, 86, 94, 153, 106, 95, 96, 89, 111, 90, 3, 154, 98, 99, 155, 101, 109, 151, 91, 112, 110, 156, 100, 159, 58, 59, 116, 60, 61, 82, 114, 128, 129, 115, 138, 140, 67, 158, 68, 81, 139, 66, 113, 130, 73, 117, 51, 55, 70, 150, 69, 54, 137, 52, 123, 119, 71, 72, 53, 56	13.883741 N, 39.162985 E / 364.77 km	682	314	1.31	13.46	< 0.001
	Secondary	250, 252, 251, 249, 248, 272, 273, 258, 253, 218, 425, 259, 506, 426, 424, 422	9.506756 N, 42.621090 E / 51.27 km	82	52	1.73	11.69	< 0.01
	Tertiary	321, 176, 315, 322, 320, 316, 323, 325, 191, 192, 377, 317, 175, 319, 318	8.245203 N, 37.785581 E / 74.06 km	106	62	1.59	10.03	< 0.05
**2005**	Primary	268, 303, 123, 337, 177, 540, 116, 450, 433, 527, 150, 335, 42, 207, 384, 348, 448, 109, 205, 346, 423, 39, 64, 162, 388, 401, 200, 530, 180, 36, 76, 188, 259, 72, 203, 195	14.108312 N, 38.288215 E/ 134.64 km	121	64	1.79	13.02	< 0.001
**2011**	Primary	191, 433, 66, 65, 133, 231, 543, 79, 469, 542, 480, 418, 224, 106, 445, 579, 463, 69, 225, 597, 296, 99, 71, 67, 226, 397, 617, 280, 322, 318, 84, 21, 482, 600, 538, 461, 164, 68, 417, 582, 235, 319, 246, 419, 589, 414, 493, 300, 510, 177, 192, 478, 241, 245, 260, 215, 287, 293, 615, 568, 497, 62, 372, 392, 311, 499, 171, 638, 512, 314, 247, 636, 533, 467, 498, 551, 366, 403, 515, 555, 604, 131, 33, 601, 195, 299, 643, 504, 521, 46, 488, 50, 217, 347, 420, 86, 577, 556, 407, 406, 35, 180, 462, 123, 119, 271, 174, 423, 334, 89, 139, 634, 20, 85, 249, 26, 219, 1, 181, 255, 77, 227, 421, 333, 388, 213, 148, 621, 557	12.635948 N, 40.297925 E / 308.64 km	880	466	1.71	67.07	< 0.001
**2016**	Primary	599, 544, 488, 344, 249, 348, 332, 241, 128, 130, 442, 427, 172, 389, 511, 79, 351, 97, 455, 449, 200, 189, 143, 571, 421, 205, 136, 191, 178, 499, 300, 392, 160, 585, 384, 496, 570, 478, 605, 235, 334, 550, 237, 94, 220, 401, 368, 424, 99, 298, 127, 611, 623, 254, 410, 362, 542, 538, 66, 591, 440, 632, 345, 196, 430, 596, 55, 263, 18, 129, 4, 134, 575, 226, 341, 547, 366, 75, 354, 192, 117, 355, 616, 579, 604, 276, 404, 617, 598, 103, 481, 199, 620, 461, 545, 627, 45, 156, 413, 628, 551, 425, 636, 84, 283, 81, 89, 590, 37, 400, 38, 460, 597, 80, 479, 135, 188, 176, 102, 340, 152, 310, 132, 584, 637, 327, 181, 322, 312, 10, 336, 267, 512, 295, 163, 456, 98, 255, 528, 39, 206, 120, 640, 158, 258, 484, 583	12.569937 N, 40.396640 E / 322.17 km	787	368	1.77	54.45	< 0.001
	Secondary	104, 260, 592, 507, 233, 69, 370, 426, 603, 346, 315, 536, 309, 567, 343, 266, 105, 106, 13, 221, 549, 417, 231, 291, 47, 469, 265, 63,114, 284, 593, 219, 270, 446	8.309769 N, 33.805118 E / 105.98 km	129	70	1.78	14.92	< 0.001
	Tertiary	138, 164, 85, 358, 146, 492, 92, 490, 543, 171, 198, 95, 318, 77, 187, 497, 556, 520, 629, 521, 588, 553, 458, 480, 208, 214, 251, 573	5.589269 N, 44.175032 E / 355.80 km	134	68	1.66	11.19	< 0.01

In EDHS 2005, a total of 36 significant the most likely clusters were identified that encompassed a mainly Tigray region, but also touched the borders of the Northern Amhara region. It was located at 14.108312 N, 38.288215 E, and 134.64 km radius. Clusters in the primary window were 1.79 times higher risk than those outside the window (RR = 1.79, LLR = 13.02, *P*-value< 0.001) (Table 5, Fig. 6 B).

During EDHS 2011, a total of 129 significant clusters, were identified in the most likely primary window. The significant cluster of the primary window encompassed mainly Eastern and Southern Tigray region Afar, Eastern and Northern Amhara, and parts of the Northern Somali region. It was located at 12.635948 N, 40.297925 E, and 308.64 km radius. Clusters in primary windows were 1.71 times higher risk than those outside the window (RR = 1.71, LLR = 67.07, *P*-value< 0.001) (Table 5, Fig. 6 C).

During EDHS 2016, a total of 208 significant clusters, were identified in three most likely SaTScan cluster areas. The most likely primary SaTScan cluster encompassed central, Eastern and Southern Tigray, Afar, and Eastern Amhara region, and some parts of the Northern Somali region. It was located at 12.569937 N, 40.396640 E, and 322.17 km radius. Clusters in the primary window had 1. 77 times higher risk than those outside the window (RR = 1.77, LLR = 54.45, *P*-value< 0.001). The second significant window included mainly the Gambela region, but also the borders of the Western Oromia region. The third most likely SaTScan cluster encompassed the most parts the Somali region and around a boundary of the Oromia region. The second and the third windows were found at 8.309769 N, 33.805118 E, and 105.98 km radius, and 5.589269 N, 44.175032 E, and 355.80 km radius, respectively. Clusters in the second and third widows were 1.78 and 1.66 higher risk of undernutrition than outside these windows (RR = 1.78, LLR = 14.92, *P*-value < 0.001 and RR = 1.66, LLR = 11.19, *P*-Value < 0.01), respectively (Table 5, Fig. 6).

### Multilevel analysis

#### Model comparison and cluster variation

Model comparison was done by using AIC. The model IV or full model with the smallest value of AIC (3514.026) was taken as the best-fitted model (Table 6).

**Table 6:** Model comparison and fitness using AIC

Model	AIC
I	3721.823
II	3561.566
III	3650.787
IV	3514.026 ^a^

^a^Best fitted model: AIC is the best fitted model (the smallest one)

The ICC value for the null model was 15.53%, which informed us to choose GLMM over the basic model (Table 7). The null model showed that undernutrition was clustered across the communities among late-adolescent girls in Ethiopia (VA^2^ = 0**.**60; *P* < 0.001).

**Table 7:** The measure of variation for under-nutrition among late adolescent girls at cluster level by multilevel binary regression analysis

Measure of variation	Model – I ^a^	*p*-value	Model-II ^b^	*P*-value	Model-III ^c^	*P*-value	Model- IV ^d^	*P*-value
Community level Variance (SE)	0.6047 (0.1057)	< 0.001	0.5934 (0.1259)	< 0.001	0.3758 (0.089)	< 0.001	0.4171 (0.109)	< 0.001
ICC %	15.53		15.28		10.25		11.25	
PCV %	Ref.		1.87		37.85		31.02	
MOR	1.78		1.76		1.43		1.49	
Model fit statistics for best fitted model								
AIC	3721.823		3561.566		3652.731		3514.026*	

*SE* standard error, *ICC* intraclass correlation, *PCV* Proportional Change in Variance, expresses the change in the cluster level variance between the null model and the individual level model, and between the individual level model and the model further including the community level covariates; *MOR* Median Odds Ratio, *DIC* Davian’s information criteria

Model I^a^ - Empty model without any explanatory variable

Model II^b^ - Adjusted for individual-level factors

Model III ^c^ - Adjusted for community-level factors

Model IV ^d^ - Full model adjusted for both individual and community-level factors

* shows the best fitted model

Intercept-only model revealed that 15.53% of the variation in the odds of late-adolescent girls’ undernutrition could be attributed to community-level factors based on the output of ICC value. The full model, after adjusting the individual and community-level factors revealed that the variation in undernutrition across the communities is statistically significant. About 11.25% of the odds of undernutrition variation across communities was observed in the full model (Table 7).

The MOR also showed that undernutrition among late-adolescent girls was attributed to community-level factors. The MOR for undernutrition was 1.78 in the intercept-only model; this indicated that there is variation between communities (clustering). The MOR value was decreased to 1.49 in model IV when we added all variables that indicated the community-level variations of undernutrition (Table 7).

The total variation attributed to individual and cluster level factors at each model was measured by the proportional change in variance (PCV) which was computed as 1.87, 37.85, and 31.02% in model II, model III and model IV respectively. This showed that there is clustering within communities that informed us to use multilevel analysis (Table 7).

#### Determinants of under-nutrition among late adolescent girls in Ethiopia

Individual and community-level factors were selected using enter methods at 0.2 significance level in bi-variable analysis.

The results of multilevel logistic regression models for individual and community level factors are shown in the table below. In the final full model, factors such as late-adolescent girls’ age, educational status, marital status and occupational status, age of household head, time to get drinking water, listening to the radio, type of toilet facility, region, and place of residence were significantly associated with late adolescent girls’ undernutrition (Table 8).

**Table 8:** Multilevel analysis of factors associated with late adolescent girls’ under-nutrition in Ethiopia, from EDHS 2016 data source

Sr.No.	Variables		Under-nutrition %		Model- I Null model	Model-II: Level –I AOR, (95%CI)	Model-III: Level-2 AOR, (95%CI)	Model-IV: L1 & L2 AOR, (95%CI)
			Yes	No				
**A.** Individual level factors								
1.	Adolescent age	15–17 years	34.30	65.70		1.00		1.00
		18–19 years	18.90	81.10		0.42, (0.32–0.55) ***		0.43, (0.33–0.53) ***
2.	Religion:	Orthodox	31.19	68.81		1.00		1.00
		Catholic	5.33	94.67		0.14, (0.03–0.73) *		0.23, (0.04–1.26)
		Protestant	23.07	76.93		0.60, (0.41–0.89) *		1.14, (0.69–1.89)
		Muslim	28.17	71.83		0.85, (0.64–1.13)		1.03, (0.70–1.51)
		Others	51.68	48.32		1.72, (0.47–6.31)		3.56, (1.03–12.24)
3.	Late Adolescent girls’ educational status	No education	27.23	72.77		1.00		1.00
		Primary	30.19	69.81		1.18, (0.81–1.72)		1.27 (0.87–1.84)
		Secondary	21.12	78.88		0.91, (0.55–1.49)		0.89, (0.54–1.47)
		Higher	40.35	59.65		4.07, (1.53–10.81) **		4.40, (1.64–11.76) **
4.	Marital status of adolescent girls	Never married	29.79	70.21		1.00		1.00
		married	23.73	76.27		1.01, (0.65–1.59)		0.95, (0.61–1.48)
		cohabited	10.57	89.43		0.35, (0.23–5.40)		0.43, (0 .03–5.76)
		Widowed	41.52	58.48		2.53, (0.08–77.16)		1.44, (0.05–40.70)
		Divorced	21.38	78.62		0.63, (0.27–1.52)		0.56, (0.23–1.36)
		Separated	17.26	82.74		0.48, (0.12–1.85)		0.46, (0.12–1.75)
5.	Occupational status of adolescents	Not working	29.70	70.30		1.00		1.00
		professional	43.51	56.49		2.33, (0.74–7.38)		2.37, (0.64–8.79)
		clerical	23.67	76.33		1.08, (0.09–13.82)		1.03, (0.07–14.28)
		sales	16.13	83.87		0.57, (0.37–0.88) *		0.60, (0.39–0.92) *
		Agri. employee	31.49	68.51		0.82, (0.55–1.23)		0.79, (0.60–1.03)
		services	24.47	75.53		0.66, (0.36–1.20)		0.73, (0.40–1.32)
		skilled manual	17.89	82.11		0.52, (0.19–1.38)		0.54, (0.20–1.45)
		Unskilled manual	43.03	56.97		1.55, (0.56–4.28)		1.42, (0.55–3.70)
		Others	33.05	66.95		1.08, (0.60–1.96)		1.07, (0.60–1.90)
6.	Age of household head	15–24	19.03	80.97		1.00		1.00
		25–34	24.22	75.78		1.20, (0.60–2.40)		1.25, (0.63–2.51)
		35–44	35.34	64.66		1.79, (0.93–3.46)		1.85, (0.97–3.53)
		45–59	30.05	69.95		1.31, (0.68–2.52)		1.31, (0.68–2.51)
		≥60	25.49	74.51		1.16, (0.59–2.28)		1.20, (0.62–2.31)
7.	Number of under five children in household	no < 5 child	25.91	74.09		1.00		1.00
		has at least one < 5 child	31.20	68.80		1.18, (0.85–1.66)		1.14, (0.81–1.58)
8.	Number of household members	1–2	21.91	78.09		1.00		1.00
		3–5	26.88	73.12		0.82, (0.46–1.47)		0.86, (0.48–1.53)
		6–7	29.39	70.61		0.87, (0.46–1.64)		0.87, (0.46–1.65)
		≥7	31.10	68.90		0.91, (0.44–1.89)		0.91, (0.44–1.87)
9.	Time to get drinking water	<30 min	31.33	68.67		1.00		1.00
		≥30 min	29.46	70.54		0.81, (0.59–1.11)		0.79, (0.58–1.09)
		Water on premises	21.67	78.33		0.81, (0.45–1.49)		0.98, (0.53–1.82)
		Others	29.00	71.00		0.60, (0.21–1.69)		0.50, (0.17–1.46)
10.	type of toilet facilities	Improved	20.67	79.33		1.00		1.00
		Un-improved	29.61	70.39		1.75, (1.13–2.72) *		1.79, (1.15–2.79) *
		Others	31.91	68.09		4.13, (0.85–21.21)		3.94 (0.65–23.95)
11.	frequency of listening to radio	Not at all	31.17	68.83		1.00		1.00
		less than once a week	24.60	75.40		0.71, (0.51–0.99) *		0.74, (0.54–1.04)
		at least once a week	21.44	78.56		0.64, (0.43–0.96) *		0.67 (0.45–0.99) *
12.	Wealth index	Poorest	32.46	67.54		1.00		1.00
		Poorer	26.61	73.39		0.62, (0.39–1.00)		0.78, (0.47–1.29)
		Middle	35.33	64.67		1.04, (0.66–1.64)		1.35, (0.83–2.19)
		Richer	29.14	70.86		0.77, (0.48–1.25)		0.99, (0.59–1.66)
		Richest	21.76	78.24		0.73, (0.40–1.35)		1.26, (0.65–2.45)
13.	type of cooking fuel	electricity	21.96	78.04		1.00		1.00
		lpg/natural gas/bio gas/ kerosene	27.45	72.55		0.79, (0.23–2.70)		0.80, (0.24–2.64)
		Charcoal	21.46	78.54		0.94, (0.55–1.61)		0.84, (0.48–1.45)
		wood	28.45	71.55		0.79, (0.45–1.36)		0.79, (0.43–1.45)
		straw/crop/animal dung	37.23	62.77		1.13, (0.54–2.35)		1.01, (0.47–2.18)
		Others	29.61	70.39		0.51, (0.08–3.16)		0.63, (0.09–4.53)
**B.** Community level variables								
**1)**	**Regions**	Tigray	43.31	56.69			1.00	1.00
		Afar	42.99	57.01			1.00, (0.68–1.46)	0.95, (0.52–1.744)
		Amhara	34.32	65.68			0.64, (0.45–0.92) *	0.56, (0.37–0.85) **
		Oromia	26.58	73.42			0.42, (0.30–0.59) ***	0.31, (0.18–0.53) ***
		Somali	45.14	54.86			1.07, (0 .72–1.60)	1.05, (0.57–1.94)
		Benishangul	20.78	79.22			0.30, (0.21–0.46) ***	0.22, (0.13–0.39) ***
		SNNPR	17.37	82.63			0.24, (0.16–0.35) ***	0.18, (0.10–0.33) ***
		Gambela	38.29	61.71			0.97, (0.58–1.6)	0.73, (0.37–1.43)
		Harari	29.98	70.02			0.70, (0.44–1.12)	0.61, (0.33–1.14)
		Addis Ababa	20.35				0.57, (0.38–0.86) **	0.58, (0.34–0.98) *
		Dire-Dawa	28.46	71.54			0.73, (0.43–1.24)	0.76, (0.38–1.56)
**2)**	Place of residence	Urban	19.51	80.49			1.00	1.00
		Rural	30.86	69.14			2.21, (1.57–3.10 ***	2.33, (1.29–4.22) **

##### Individual-level factors

The odds of undernutrition among 18–19 years of adolescent girls, was 57% (AOR = 0.43; 95% CI: (0.35–0.53) lower than those 15–17 years old. Late-adolescent girls with higher educational status were 4.40 times (AOR = 4.40; 95% CI: (1.64–11.76) more likely to be undernourished than those with no educational status. The odd of undernutrition among late-adolescent girls, with the occupation of sales, was 40% (AOR = 0.60; 95% CI: 0.43–0.84) lower than those with not working adolescents. The odds of undernutrition, among late-adolescent girls, having an unimproved latrine type, was 1.79 times (AOR = 1.79; 95% CI: 1.15–2.79) higher than those participants with improved latrine type. Participants with the frequency of listening to the radio at least once a week were 33% (AOR = 0.67; 95% CI: (0.45–0.99) less likely to be undernourished than those without listening to the radio (Table 8).

##### Community-level factors

The odds of late-adolescent girls’ undernutrition, in the regions of Amhara, Oromia, Benishangul Gumuz, SNNPR, and AA, was 44% (AOR = 0.56; 95% CI: 0.37–0.85), 69% (AOR = 0.31; 95% CI: 0.18–0.53), 78% (AOR = 0.22; 95% CI: 0.13–0.39), and 82% (AOR = 0.18; 95% CI: 0.10–0.33), and 42% (AOR = 0.58, (95% CI: 0.34–0.98) lower compared with Tigray region, respectively. The odds of undernutrition among late-adolescent girls with rural residents was 2.33 times higher (AOR = 2.33; 95% CI: 1.29–4.22) than those with urban residents (Table 8).

## Discussion

The current study showed that the prevalence of undernutrition among late-adolescent girls was decreased overtime in the past 15 years in Ethiopia. It had decreased from 36.79, 95% CI: (34.11–39.54) in EDHS 2000 to 28.22, 95% CI: (26.02–30.52%) in EDHS 2016. This could be due to the fact that the Ethiopian government gave attention to the improvement of agricultural production and productive safety net programs. However, there were still regional variations of undernutrition in Ethiopia. Four regions, namely, Tigray, Afar, Amhara, and Somali regions had a higher prevalence of undernutrition (greater than the national average) during each survey period, while Addis Ababa, SNNPR, and Oromia regions were in the lowest prevalence in each survey. This might be due to the fact that the decreased amount of rainfall distribution and recurrent attacks of drought in the Northern and Eastern parts of the country affected agricultural products and sources of food for animals.

Undernutrition was spatially clustered nationally during each survey period of the study. The spatial distribution of high prevalence undernutrition in each survey period was mainly in Northern, Eastern, and some Western parts of the country, including Tigray, Afar, Eastern Amhara, Harari, Somali, and Gambela regions. These variations might be because of recurrent attacks of drought, climate changes and decreased rainfall distributions, land degradation, and soil erosion for crop production that happened in the above regions of Ethiopia in different time periods.

In this study, the local spatial distribution showed that the spatial variation of undernutrition in different parts of Ethiopia. In EDHS 2000, the statistically significant hot spot areas of undernutrition, among late-adolescent girls, was observed in the regions of central and eastern Tigray, Southern Afar, Eastern Amhara, Southwest and Southern Oromia, Southern and Northern Somali region, and SNNPR, whereas in the 2005 survey, the hot spot areas of undernutrition were observed in Tigray, Western borders of Afar, Northern and Eastern Amhara, and the Southwestern Somali region. This might be related to seasonal attacks of drought and decreased rainfall distributions from these areas that could result in difficulties in crop production and limited food security. Likewise, in the EDHS 2011, the hot spot areas of undernutrition were observed mainly in Tigray, Western and Eastern Afar, Northern and Eastern Amhara, Southwestern Somali region, and Gambela region, while During 2016 survey, the statistically significant hot spot areas were observed in the regions of Tigray, Northern and Eastern Amhara, Afar, Benishangul-Gumuz, Somali region, and Gambela region. This might be attributed to recurrent seasonal attacks of drought in the Northern, Eastern, and Western parts of the country. Agriculture is mainly affected by climate changes in Ethiopia [27], that impacted food security. In 2015, the El Niño drought, which was one of the strongest droughts in Ethiopia that caused more than 27 million people became food-insecure which is one of the sources of undernutrition [4].

This study also revealed that the highest prediction of undernutrition prevalence was observed in the different regions in different time periods. In EDHS 2000, the highest prediction of undernutrition, among late-adolescent girls, from un-sampled EAs areas, was observed in the regions of Tigray, Afar, Eastern Amhara, Southwestern, and Southeastern Oromia, Southwestern Somali, and NNPR. However, EDHS 2005 revealed that the highest prediction of undernutrition, among late-adolescent girls, from un-sampled enumeration areas, was observed in the regions of Tigray, Northern Afar, Northern and Eastern Amhara, and Southern Somali region. Similarly, EDHS 2011 remarked that the highest risk of undernutrition, among late-adolescent girls, from un-sampled cluster areas, was predicted in Tigray, Afar, Amhara, Southern Somali, and parts of Gambela regions. Likewise, during EDHS 2016, the highest prediction of undernutrition risk, among late-adolescent girls, from un-sampled enumeration areas, were identified in Tigray, Afar, Amhara, Somali region, and Gambela regions.

This study showed that a number of statistically significant clusters with high and low rates were mainly observed in the Northern and Eastern parts of Ethiopia during each survey period. In EDHS 2000, there were a total of 153 statistically significant clusters detected, in three most likely SaTScan windows with a high prevalence of undernutrition that encompassed Tigray, Afar, Amhara, Southwest Oromia, and Somali regions, whereas during the 2005 survey, a total of 36 significant clusters, were identified in the primary SaTScan cluster area, which encompassed mainly the Tigray region. Likewise, during EDHS 2011, a total of 129 significant clusters with a high prevalence of undernutrition were identified in the most likely SaTScan primary cluster that encompassed mainly the Eastern and Southern Tigray region, Afar, Eastern Amhara and Northern Somali region. During EDHS 2016, a total of 208 significant clusters, were identified in the three most likely SaTScan cluster areas. The most likely SaTScan primary window encompassed central, Eastern, and Southern Tigray, Afar, and Eastern Amhara regions and the Northern Somali region. The second significant window included Gambela, and Western Oromia regions and the third SaTScan window encompassed the most parts of the Somali region and around a boundary of the Oromia region. These high rates of undernutrition with significant clusters in different regions may be attributed to because 2015 El-Nino drought that affected food securities in the above regions because of significant rainfall decrement and many livestock deaths in Afar, Somali, and Oromia pastoralists [4, 28].

The current study identified that the regional variation of undernutrition, among late-adolescent girls, was attributed to both individual and community level factors. In the final full model, individual and community level factors accounted for about 31.02% of the variations for undernutrition among late-adolescent girls. Multilevel analysis of this study showed that different individual and community-level factors influenced undernutrition among late-adolescent girls in Ethiopia.

In the final full model, the age group of late-adolescent girls was significantly associated with undernutrition. The odds of undernutrition, among 18–19 years of adolescent girls, was 57% less likely than those 15–17 years old. This may be because of the synergistic effect of growth velocity during puberty when peak height velocity occurs and endocrine factors are also essential for promoting normal adolescent growth and are sensitive to undernutrition [12]. This was supported by a study in Northwestern Ethiopia [29], the age groups of 15–17 years were 2 times higher for being undernourished than 18–19 years old adolescents. On the other hand, adolescent girls after 18 years may be engaged in marriage and may be better access to eating patterns in economically limited families. In contrast with the above, a study from India [30] showed that late-adolescents were less likely for undernutrition compared with early-adolescents.

The current study identified that late-adolescent girls with higher educational status were 4.40 times more likely to be undernourished compared with no educational status. This may be attributed to girls are more vulnerable to the influences of cultural and gender norms, which often discriminate against frequent feeding and when dietary intakes are suboptimal, anemia and micronutrient deficiencies are high among adolescent girls [12]. On the other hand, during higher education, those adolescents may go to places far from their parents, so that they are limited to get timely feeding and food varieties are limited due to economic barriers. A study stated that eating patterns and behaviors are influenced by peer pressure, food availability, food preferences and cost, personal, and cultural beliefs [1]. A study from low and middle-income countries also remarked that about 40% of adolescent girls reported skipping their breakfast [31].

This study identified that late-adolescent girls with the occupation of sales were 40% lower for being undernourished than those with not working. This might be because they have their own money who easily purchase varieties of food items and fulfil their own food security.

This study stated that the odds of undernutrition among participants with unimproved latrine type was 1.79 times higher than from participants with improved latrine type. This may be attributed to poor sanitation could expose infestation of intestinal parasites that leads to illness, poor appetite and micronutrient deficiencies that leads to undernutrition. This was consistent with the SRMA study in Ethiopia [6].

Participants with the frequency of listening to the radio at least once a week were 33% less likely to be undernourished than those without listening to the radio. This could be because of better awareness and information gain regarding the importance of a variety of food items, and frequency of feeding patterns among those listening to the radio.

In the current study, the region as a factor was significantly associated with undernutrition. The odds of undernutrition, among late-adolescent girls, in the regions of Amhara, Oromia, Benishangul Gumuz, SNNPR, and AA, was 44, 69, 78, 82 and 42% lower compared with the Tigray region, respectively. This might be because of divergence to access food security across regions in Ethiopia due to seasonal climate changes, rainfall distributions, and soil degradation and erosion for crop production [4]. This was supported by a study done in the Amhara region that showed adolescent girls living in food-secured households were 35% less likely to be undernourished than their counterparts [29]. Moreover, an SRMA study in Ethiopia showed that adolescent girls, among food-insecure households, were 2.38 times higher for being short stature than food-secure families [6].

This study revealed that the odds of undernutrition among late-adolescent girls with rural residents was 2.33% times higher compared with urban residents. This was consistent with a recent SRMA study in Ethiopia that remarked, being the rural residence was 2.19 times higher for being undernourished than urban residents [6]. This might be attributed to cultural influences, lack of awareness about the importance of a variety of foods, and due to food insecurity in rural areas because of recurrent drought attacks, climate changes and land degradation, and soil erosion [4]. It was supported by a study in the Northwestern Amhara region [8] which stated that the odds of stunting was 45% higher among adolescents of rural areas with food-insecure households. On the other hand, a systematic review study done among adolescent girls in low- and middle-income countries showed that the mean energy intake was lower in rural settings compared to urban settings [31].

### Strength and limitation of the study

Since we used four consecutive large data sets of EDHS, the study was nationally representative. Spatial analysis was used to explore hot spots and high-rate significant cluster analysis that would be important to design interventions at the community level. Multilevel analysis was used to account for the cluster-level effect of correlations that helped for a better estimate of the level of association. Since it was secondary data analysis, we didn’t find some important variables, such as food security, a variety of foods, and clinical related variables, and variables like husband educational status and occupational status were not consistently collected in each survey.

## Conclusion

The current study found that undernutrition among late-adolescent girls was clustered across regions in Ethiopia in each survey. The spatial patterns of this study showed that there was a high spatial dependency across regions. The spatial scan statistics revealed that the significant clusters with a high prevalence of undernutrition encompassed Northern, Eastern, and also Western parts of Ethiopia recently. Age groups, educational status and occupational status of adolescents, unimproved latrine type, frequency of listening to the radio, household head, being rural residents, and region were significantly associated with undernutrition. Therefore, the government of Ethiopia and stakeholders should take responsibility in these areas to intervene in undernutrition early. Awareness creation via mass media and health education regarding importance of using an improved latrine type is mandatory.

## Data Availability

The availability of data for this particular study was from the DHS program datasets using the website **www.measuredhs.com****.** after we had sent the research question.

## Abbreviations

AIC: Akaike’s Information Criterion
AOR: Adjusted Odds Ratio
ArcGIS: Aeronautical Reconnaissance Coverage Geographic Information System
BMI: Body Mass Index
DALIYs: Disability Adjusted Life Years
DHS: Demographic Health Survey
EAs: Enumeration Areas
EDHS: Ethiopian Demographic and Health Survey
GLMM: Generalized Linear Mixed Model
HH: Household
ICC: Intra-class Correlation Coefficient
IQR: Inter Quartile range
LLR: Log-Likelihood Ratio
LBW: Low Birth Weight
MCH: Maternal and Child Health
MOR: Median Odds Ratio
PCV: Proportional Change in Variance
SD: Standard Deviation
SNNPR: South Nations Nationalities and Peoples Representatives
TB: Tuberculosis
VA: variance of the initial model
VB: is the variance of the model with more terms
